# Effects of liquid diet administration routes and types of dietary fiber pectin on fecal characteristics and gut microbiota in rats

**DOI:** 10.3389/fnut.2026.1812566

**Published:** 2026-06-08

**Authors:** Tomohiro Ikeda, Sho Miyatake, Ryosuke Akiyama, Ippei Yamaoka

**Affiliations:** Medical Foods Research Laboratory, Otsuka Pharmaceutical Factory, Inc., Naruto, Japan

**Keywords:** duodenostomy, gastrostomy, gut microbiota, liquid diet, pectin, percutaneous endoscopic gastrostomy

## Abstract

**Introduction:**

Enteral nutrition is essential for the nutritional management of patients with feeding or swallowing difficulties; however, the effects of different administration routes and dietary fiber types on the gut environment are insufficiently understood. This study aimed to compare the effects of enteral feeding route and pectin type on physiological parameters, fecal characteristics, and gut microbiota composition.

**Methods:**

Male Sprague–Dawley rats (total *n* = 81) received liquid diets differing in administration routes (oral, gastrostomy, and duodenostomy) and pectin type [low-methoxyl pectin [LMP] and high-methoxyl pectin (HMP), or no pectin]. Outcomes included cecal content weight, plasma biomarkers, and gut microbiota assessed by 16S rRNA sequencing.

**Results:**

Compared with the gastrostomy group, the duodenostomy group increased cecal content weight, reduced gut microbiota *α* diversity, and shifted microbial composition toward decreased Firmicutes and increased Proteobacteria. The duodenostomy group also showed alterations in inflammation and dysbiosis-associated bacterial taxa, including decreased Lachnospiraceae and increased Enterobacteriaceae, accompanied by elevated portal vein endotoxin and indole concentrations. In contrast, LMP-supplemented liquid diets improved fecal characteristics irrespective of administration route and were associated with increased *α* diversity, increased Firmicutes, and reduced Enterobacteriaceae, suggesting a potential corrective effect on enteral nutrition-induced alterations in the gut environment.

**Conclusion:**

These findings suggest that the route of liquid diet administration in this short-term rat model is associated with differences in intestinal environment, and that these associations vary according to dietary fiber type. Dietary fiber type was also associated with the maintenance of the gut environment and stool characteristics, providing experimental evidence that may contribute to future translational research.

## 1 Introduction

Nutritional management in clinical practice is a critical determinant of patient prognosis and quality of life, particularly for individuals who are unable to ingest food orally and rely on liquid diets as their main source of nutrition. Liquid diets may be administered via several routes, including nasogastric tubes, gastrostomy, and intestinal stomas such as jejunostomy and duodenostomy. The route is selected according to the patient’s underlying disease and gastrointestinal status. Enteral nutrition confers several benefits to the gut, including preservation of immune function, maintenance of intestinal barrier integrity, and prevention of infectious complications (1). Conversely, gastrointestinal symptoms associated with enteral nutrition, such as impaired intestinal absorption and diarrhea, increase the risk of malnutrition, acidosis, and pressure ulcers, thereby complicating nutritional management and potentially increasing morbidity and mortality (2).

In recent years, the effects of enteral feeding routes on the gut environment have received increasing attention. Patients receiving enteral nutrition have been reported to show reduced gut microbiota diversity and changes in the abundance of specific gut bacteria (2–4). For instance, fecal analyses have demonstrated a reduction in anaerobic bacteria and a concomitant increase in aerobic bacteria, accompanied by a substantial decrease in the number of culturable bacterial strains (5). Alterations in the gut microbiota during enteral nutrition are influenced by multiple factors, including the macronutrient composition of liquid diets (particularly protein content) (6, 7), the level of dietary fiber supplementation (3, 8), and the use of medications such as antiepileptic drugs and antibiotics (3). However, few studies have directly compared gut microbiota changes associated with different administration routes, and existing studies (3, 9) are limited by the lack of standardized diet formulations and small sample sizes. Consequently, the long-term effects of different enteral feeding routes on the gut environment, and their clinical significance, remain poorly understood, including the effects of duodenostomy feeding.

Dietary fiber intake helps maintain gut microbiota diversity, enhances short-chain fatty acid (SCFA) production (10), reduces diarrhea (11, 12), and improves bowel function (13). These effects depend not only on the total amount of dietary fiber but also on fiber type and physicochemical properties. Generally, insoluble dietary fibers primarily contribute to fecal bulk and stimulation of intestinal peristalsis, whereas soluble dietary fibers are more readily fermented by the gut microbiota and contribute mainly to SCFA production and modulation of the gut environment (14). Incorporating dietary fiber into liquid diets may support gut microbiota diversity, intestinal barrier function, and immune homeostasis through enhanced SCFA production (15). In severely ill children receiving enteral nutrition via nasogastric feeding or gastrostomy, dietary fiber intake is often reduced to approximately two-thirds of the recommended level, which may contribute to dysbiosis (16). However, an optimal, route-specific composition of dietary fiber type and content in liquid diets has not yet been established.

Pectin is a water-soluble dietary fiber extracted from citrus fruits such as lemons and limes and consists of a linear polymer of galacturonic acid residues that are partially methyl-esterified at the carboxyl groups. Based on the degree of esterification, pectin is commonly classified as low-methoxyl pectin (LMP; <50%) or high-methoxyl pectin (HMP; ≥50%), which differ in gelling behavior and the physical properties of the resulting gels. LMP undergoes gelation through cross-linking mediated by divalent cations such as calcium, particularly under low-pH conditions like those in the stomach (17, 18). Through this mechanism, LMP-containing liquid diets have been reported to increase luminal viscosity and alter transit time in the upper gastrointestinal tract, while the water-holding capacity associated with gel formation helps maintain normal fecal characteristics (17). Furthermore, LMP feeding has been reported to significantly increase SCFA production in the rat cecum (19), suggesting that the chemical structure of pectin plays an important role in intestinal fermentation.

Based on these considerations, we hypothesized that the route of liquid diet administration and the type of pectin would exert distinct effects on the gut environment. Accordingly, this study examined the main effects and interaction between administration route (oral, gastrostomy, and duodenostomy) and pectin type [LMP, HMP, or pectin-free (PF)] on the gut environment in rats.

## 2 Materials and methods

This study was conducted in accordance with the animal experimentation guidelines of Otsuka Pharmaceutical Factory, Inc. (Tokushima, Japan) and was approved by the Animal Experiment Ethics Committee of Otsuka Pharmaceutical Factory (approval number: OPFCAE-2024120).

### 2.1 Physicochemical measurements of the experimental diets

Energy, protein, carbohydrates, dietary fiber, LMP, HMP, calcium, lipids, and PFC are listed as theoretical values based on the respective ingredient proportions. Viscosity was measured using a B-type viscometer (L rotor, 12 rpm, 25 °C) after sterilization treatment and allowing the sample to equilibrate at room temperature (25 °C) for 1 to 5 days. Osmolality was determined using the freezing point depression method, following the “Osmotic Pressure Measurement Method (Osmolality Measurement Method)” of the Japanese Pharmacopoeia. Measurements were performed using an “OSMETTE XL (Relate Co., Ltd.),” with 2 mL of the sample pre-equilibrated to 25 °C.

### 2.2 Laboratory animals and test design

Seven-week-old male Crl: CD(SD) rats (total *n* = 81) obtained from Jackson Laboratory Japan Co., Ltd., Yokohama, Japan, were used. Animals were housed at 23 ± 3 °C and 55 ± 15% relative humidity under a 12-h light–dark cycle (7:00 a.m. to 7:00 p.m.). They were acclimated for 18 days and provided *ad libitum* access to a standard diet (AIN-93G; Oriental Yeast Co., Ltd., Tokyo, Japan) and water throughout the experimental period. During acclimation, cage mates were reassigned every 3 days to equilibrate the gut microbiota among animals. One day before surgery, animals were randomly assigned to the oral, gastrostomy, or duodenostomy groups (*n* = 18 per group). Consistent with our pilot findings, the required sample size was calculated *a priori* using the effect size estimated from preliminary data, with statistical power set at 0.80 and a two-sided *α* of 0.05, yielding *n* = 3. All animals underwent 24 h of preoperative water withdrawal and 48 h of fasting to improve surgical tolerance and ensure gastrointestinal emptying. Before surgery, body weight was measured for all animals, and they were divided into nine groups. Any animals exhibiting tube occlusion or clinical deterioration were humanely euthanized under isoflurane anesthesia. Stratified randomization based on body weight was conducted using the statistical analysis system Stat PreClinica (Takumi Information Technology Inc.). Gastrostomy and duodenostomy were performed as described below. Under isoflurane anesthesia (MSD Animal Health, Tokyo, Japan), an approximately 2-cm midline abdominal incision was made just caudal to the sternum to expose the stomach. A small opening was created in the gastric fundus, and the tip of a 6.5-Fr Kangaroo™ enteral feeding tube (Cardinal Health™, Ohio, USA) was inserted. In the gastrostomy group, the tube was advanced into the stomach through the gastric puncture site. In the duodenostomy group, the tube was advanced from the gastric fundus through the pylorus into the duodenum. The area around the insertion site was sutured with 3–0 suture (Ethicon, Inc., New Jersey, United States) to secure the tube. The opposite end of the tube was passed through the abdominal wall and tunneled subcutaneously to the dorsal region. An approximately 5-mm incision was made in the interscapular region to exteriorize the tube. The abdominal muscle layer was sutured with 6–0 sutures (Ethicon, Inc.), and the skin was closed with surgical staples. In the oral group, an approximately 2-cm skin incision was made along the abdominal midline and subsequently closed with sutures. Postoperatively, all animals received a single intramuscular injection of Viccillin (5 mg per animal, Meiji Seika Pharma Co., Ltd., Tokyo, Japan) into the right hindlimb. Correct placement of the tube tip was confirmed in all animals at necropsy. Viccillin (Benzathine penicillin G) is a depot formulation that yields low but sustained systemic concentrations after deep intramuscular administration. Given its narrow antimicrobial spectrum, it is expected to exert a limited effect on the gut microbiota (20).

**Figure 1:**
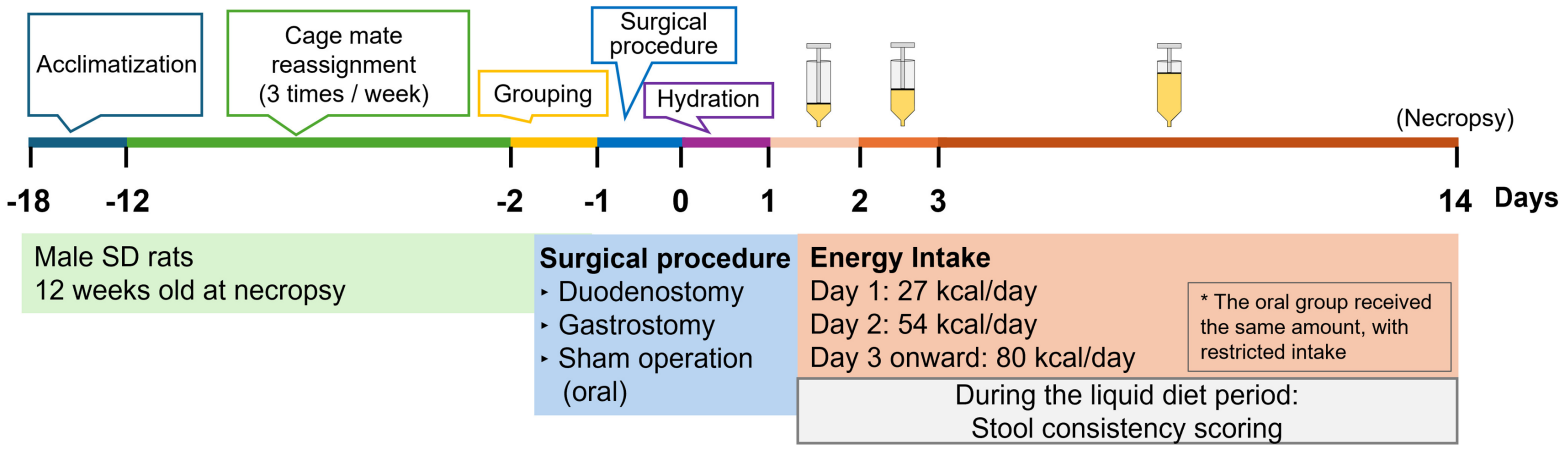
Experimental scheme. Schematic diagram of the study timeline. Type of rat used, treatment method, amount of energy administered, and administration period.

Beginning the day after placement at 4:00 p.m., liquid diets containing LMP, HMP, or no pectin were administered for 14 days to the oral groups (Oral-LMP, *n* = 6; Oral-HMP, *n* = 6; Oral-PF, *n* = 6), gastrostomy groups (Gas-LMP, *n* = 6; Gas-HMP, *n* = 6; Gas-PF, *n* = 6), and duodenostomy groups (Duod-LMP, *n* = 6; Duod-HMP, *n* = 6; Duod-PF, *n* = 6). Each liquid diet was prepared based on the formulation of a commercially available pectin-containing liquid diet (HINEX E-Gel; Otsuka Pharmaceutical Factory, Tokushima, Japan), as summarized in Table 1. Liquid diets were administered as a continuous 24-h infusion via the gastrostomy or duodenostomy route, with stepwise increases in energy provision (Day 1: 27 kcal/day; Day 2: 54 kcal/day; Day 3 onward: 80 kcal/day). The oral group received an isocaloric liquid diet under restricted feeding conditions equivalent to the intake of the gastrostomy and duodenostomy groups (Figure 1). The oral intake group was administered the diet intermittently and was designed to serve as a physiological model. All animals were deprived of drinking water during the liquid diet period. However, the test diet contained 1.1 mL/kcal; thus, administration of 80 kcal per day from day 3 onward provided 88 mL/day of fluid, which exceeds the estimated daily water requirement for rats weighing 350–400 g, making dehydration during this period unlikely. On the final day of administration, body weight was recorded, blood samples were collected from the abdominal aorta and portal vein under isoflurane anesthesia, and cecal contents were harvested.

**Table 1:** Composition of the experimental diets.

Physical properties of the samples		PF liquid diet	HMP liquid diet	LMP liquid diet
Energy	(kcal/mL)	0.8	0.8	0.8
Protein	(g/100 kcal)	4.0	4.0	4.0
Carbohydrates	(g/100 kcal)	15.4	16.8	16.8
Water content	(mL/100 kcal)	110	110	110
Dietary fiber	(g/100 kcal)	0.5	1.4	1.4
LMP	(g/100 kcal)	0	0	0.9
HMP	(g/100 kcal)	0	0.9	0
Calcium	(mg/100 kcal)	58.8	58.8	58.8
Fat	(g/100 kcal)	2.2	2.2	2.2
P:F:C	—	16:20:64	16:20:64	16:20:64
Viscosity	(mPa s)	5	5	10
Osmotic pressure	(mOsm/L)	341	371	360

LMP, Low methoxyl pectin; HMP, High methoxyl pectin; PF, Pectin free; P, Protein; F, Fat; C, Carbohydrate.

### 2.3 Blood analysis

Blood samples (*n* = 6/group) were collected into lithium-heparin tubes and immediately chilled on ice. Plasma was obtained by centrifugation at 4 °C and 3,000 rpm for 10 min. Plasma collected from the abdominal aorta was subjected to biochemical analyses, which were outsourced to Oriental Yeast Co., Ltd., Nagahama Life Science Laboratory (Shiga, Japan).

### 2.4 Stool consistency scoring

During the liquid diet period, fecal consistency was assessed daily according to previously reported criteria (17): normal stool = 0, soft stool = 1, pasty stool = 2, and watery stool = 3. Scores were calculated by dividing the number of fecal samples assigned to each score by the total number of fecal outputs for each animal (*n* = 6/group). The mean value over the entire administration period was then determined.

### 2.5 16S ribosomal RNA (rRNA)-based analysis of cecal content microbiota

Cecal contents (*n* = 6/group) were snap-frozen in liquid nitrogen immediately after collection and stored at −80 °C until analysis. To analyze microbiota composition, 16S rRNA sequencing was performed by TechnoSuruga Laboratory (Shizuoka, Japan). DNA extraction and PCR amplification were carried out according to Takahashi *et al.* (21). The V3-V4 variable region of the 16S rRNA gene was amplified from bacterial genomic DNA by PCR using DNA extracts as templates and the primers 341F (5′-CCTACGGGAGGCAGCAG-3′) and 805R (5′-GACTACCAGGGTATCTAATC-3′). After quality assessment and purification, amplicon sequencing was performed on the MiSeq platform (Illumina, San Diego, CA, United States). Sequencing data were generated in FASTQ format, and downstream analyses were outsourced to Bioengineering Lab. Co., Ltd. (Kanagawa, Japan). Amplicon sequence variants (ASVs) were inferred using the DADA2 plugin in QIIME 2 (version 2024.2), with chimeric and erroneous sequences removed. Taxonomic classification was performed using the feature-classifier plugin in QIIME 2 (version 2024.2) by comparing representative sequences with the SILVA reference database (version 138; 99% identity). Alpha diversity was calculated using the Chao1 (22) and Shannon (23) indices. Beta diversity was visualized by principal coordinates analysis (PCoA) based on weighted UniFrac distances (24). All diversity analyses and PCoA were performed using the QIIME 2 diversity plugin (version 2024.2).

### 2.6 Measurement of plasma endotoxin and indole

Endotoxin and indole concentrations were measured in portal vein plasma (*n* = 6/group). Endotoxin levels were determined colorimetrically using a Limulus amebocyte lysate (LAL) chromogenic endpoint assay kit (HIT302; Hycult® Biotech, Netherlands), according to the manufacturer’s instructions. Indole concentrations in portal vein plasma were measured colorimetrically using an indole assay kit (DIND-100; BioAssay Systems, California, United States), also following the manufacturer’s instructions. All measurements were performed in duplicate.

### 2.7 Statistical analysis

Two-way analysis of variance (ANOVA) was performed with administration route (oral, gastrostomy, duodenostomy) and pectin type (LMP, HMP, PF) as factors, including interaction effects. When a significant interaction was detected (*p* < 0.05), *post hoc* comparisons were conducted to evaluate simple effects (i.e., group differences within each level of the other factor) using Tukey’s HSD multiple comparison test. Results are presented as mean ± standard deviation, with statistical significance set at *p* < 0.05. Analyses were performed using Statistical Analysis System version 9.4 (SAS Institute, Cary, NC, United States).

## 3 Results

### 3.1 Body weight, blood biochemistry, and plasma ammonia concentration

**Figure 2:**
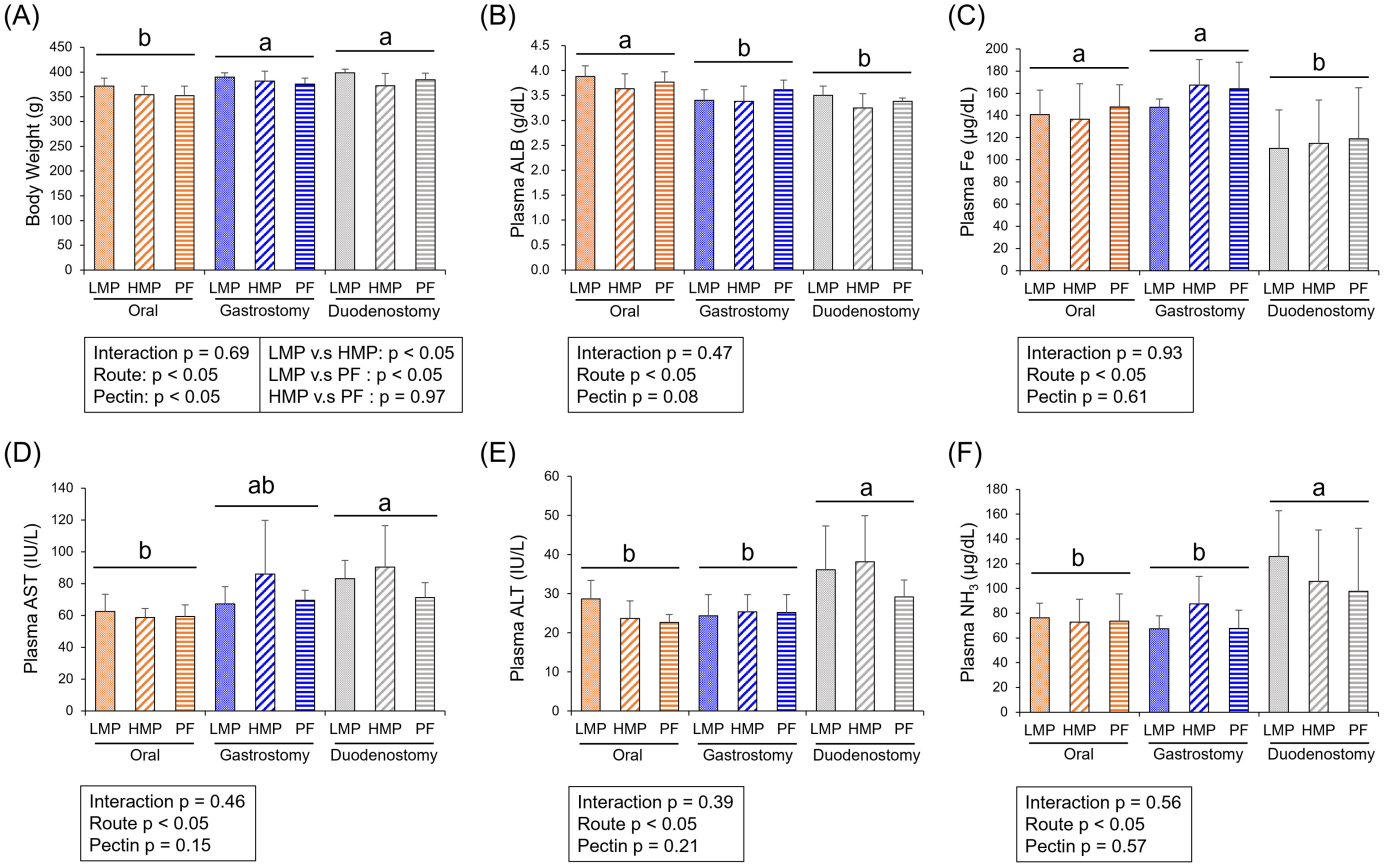
Body weight and blood biochemical markers. Liquid diets containing low-methoxyl pectin (LMP), high-methoxyl pectin (HMP), and no pectin (PF) were administered for 14 days via oral, gastrostomy, and duodenostomy routes, respectively. **(A)** Body weight, **(B)** Plasma albumin concentration, **(C)** Plasma iron (Fe) concentration, **(D)** Plasma AST concentration, **(E)** Plasma ALT concentration, **(F)** Plasma ammonia (NH_3_) concentration. Data are presented as mean ± standard deviation (SD) (*n* = 6 per dietary condition, *n* = 18 per administration route group or pectin group). When significant main effects or interaction effects were observed, the means were compared using Tukey’s HSD multiple comparison test with a *p* < 0.05 indicating significance. Different letters (a, b, and c) indicate significant differences among the oral, gastrostomy, and duodenostomy groups (*p* < 0.05). Symbols of *p*-value of route main effect and pectin main effects (LMP, HMP, and PF) are illustrated at the bottom of each panel.

Final body weight was significantly higher in the gastrostomy and duodenostomy groups than in the oral group (Figure 2A; main effect of administration route, *p* < 0.05). Body weight was also significantly greater in the LMP groups than in the HMP and PF groups, regardless of administration route (Figure 2A; main effect of pectin type, *p* < 0.05). Plasma albumin levels were significantly lower in the gastrostomy and duodenostomy groups than in the oral group (Figure 2B; main effect of administration route, *p* < 0.05). Plasma iron (Fe) concentrations were significantly lower in the duodenostomy group than in the oral and gastrostomy groups (Figure 2C; main effect of administration route, *p* < 0.05). Plasma aspartate aminotransferase (AST) levels were significantly higher in the duodenostomy group than in the oral group (Figure 2D; main effect of administration route, *p* < 0.05). Plasma alanine aminotransferase (ALT) levels were also significantly higher in the duodenostomy group than in the oral and gastrostomy groups (Figure 2E; main effect of administration route, *p* < 0.05). Plasma ammonia (NH_3_) concentrations were significantly higher in the duodenostomy group than in the oral and gastrostomy groups (Figure 2F; main effect of administration route, *p* < 0.05). In contrast, for plasma iron (Fe), AST, ALT, and ammonia concentrations (Figures 2C–F) did not differ significantly between the oral and gastrostomy groups.

### 3.2 Fecal characteristics and cecal content weight

**Figure 3:**
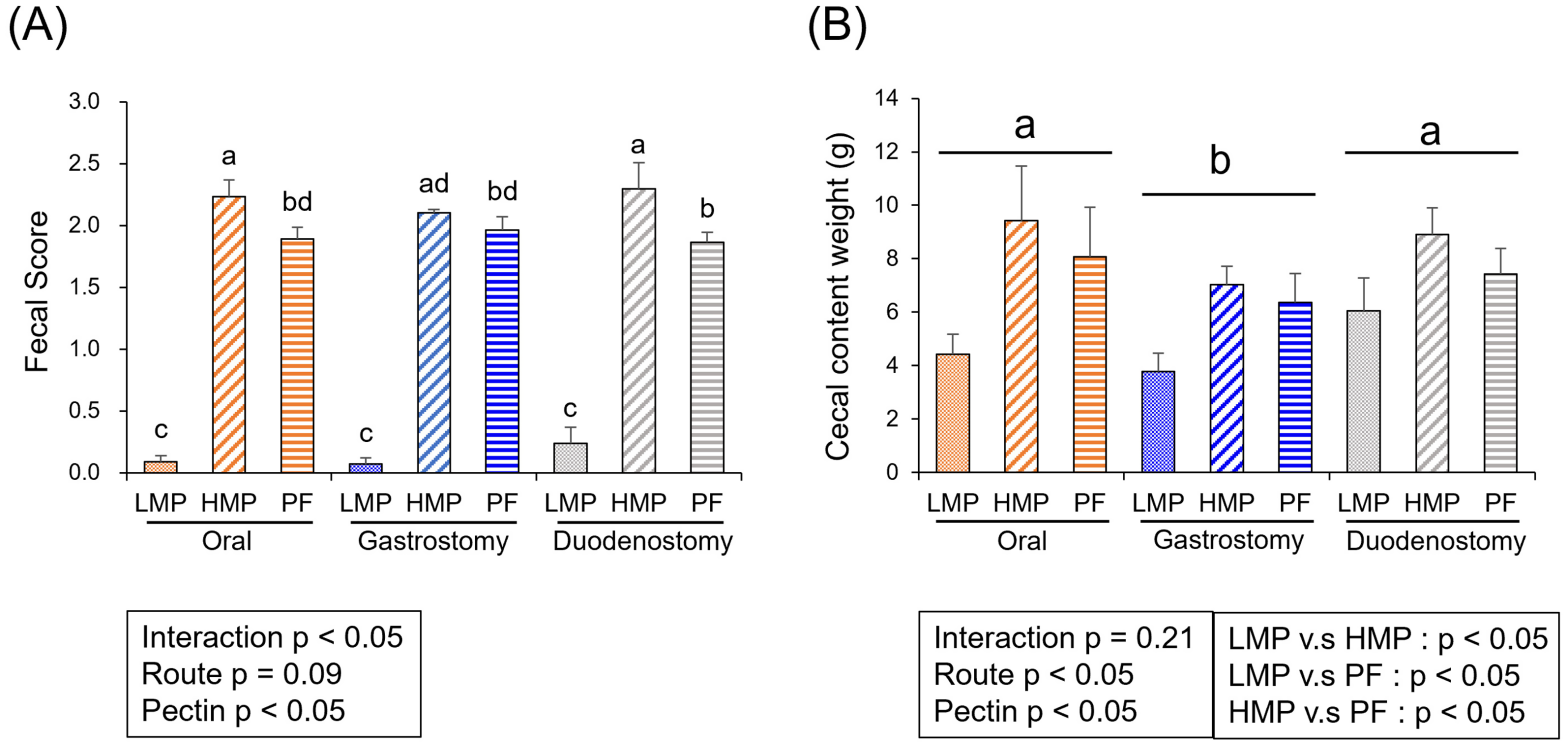
Fecal characteristics and cecal content weight. **(A)** Stool consistency score (mean score during the feeding period), **(B)** Weight of cecal contents. The stool consistency score was evaluated as follows: normal stool: 0 points, soft stool: 1 point, pasty stool: 2 points, watery stool: 3 points. Data are presented as mean ± standard deviation (SD) (*n* = 6 per dietary condition, *n* = 18 per administration route group or pectin group). When significant main effects or interaction effects were observed, the means were compared using Tukey’s HSD multiple comparison test with a *p* < 0.05 indicating significance. Different letters (a, b, c, and d) indicate significant differences between groups when an interaction is present, and significant differences among the oral, gastrostomy, and duodenostomy groups when no interaction is present (*p* < 0.05). Symbols of p-value of route main effect and pectin main effects (LMP, HMP, and PF) are illustrated at the bottom of each panel.

Figure 3A presents the mean fecal consistency score across the administration period. A significant interaction between administration route and pectin type was observed (*p* < 0.05). Across all routes, fecal consistency scores were significantly lower in the LMP groups than in the HMP and PF groups (interaction effect, *p* < 0.05).

Cecal content weight was significantly higher in the oral and duodenostomy groups than in the gastrostomy group (Figure 3B; main effect of administration route, *p* < 0.05). It was also significantly higher in the HMP and PF groups than in the LMP group (Figure 3B; main effect of pectin type, *p* < 0.05).

### 3.3 Effect of administration route and pectin type on cecal microbiota composition

**Figure 4:**
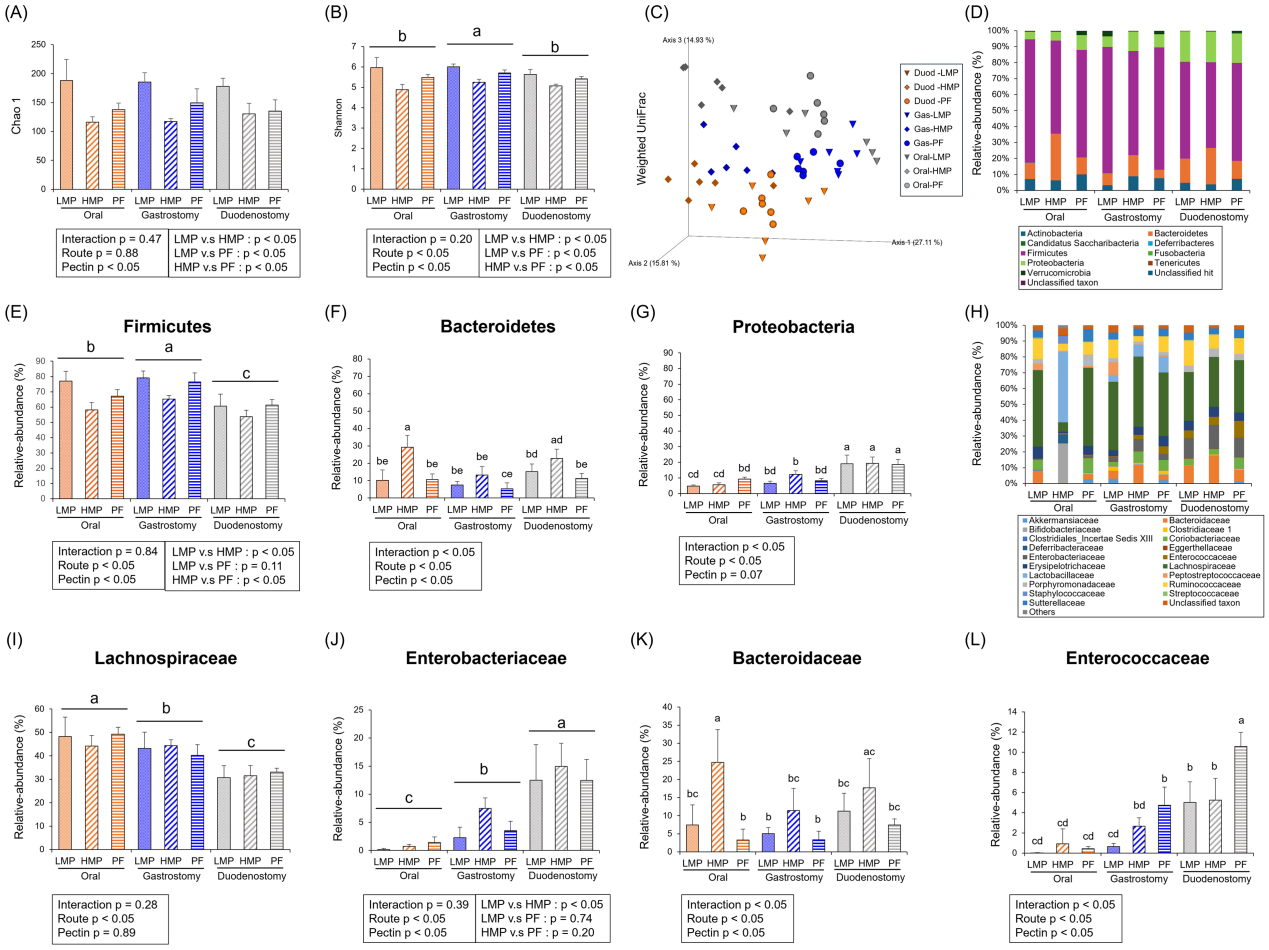
Effects of administration route and pectin type on the diversity and composition of the bacterial flora in cecal contents. *α* diversity analysis using **(A)** the Chao1 index and **(B)** the Shannon index. **(C)** *β* diversity analysis using principal coordinates analysis (PCoA) based on weighted UniFrac distance. **(D)** Comparison of relative abundance at the phylum level: **(E)** Firmicutes, **(F)** Bacteroidetes, **(G)** Proteobacteria. **(H)** Comparison of relative abundance at the family level: **(I)** Lachnospiraceae, **(J)** Enterobacteriaceae, **(K)** Bacteroidaceae, **(L)** Enterococcaceae. Data are presented as mean ± standard deviation (SD) (*n* = 6 per dietary condition, *n* = 18 per administration route group or pectin group). When significant main effects or interaction effects were observed, the means were compared using Tukey’s HSD multiple comparison test (*p* < 0.05). Different letters (a-e) indicate significant differences between groups when an interaction is present, and significant differences among the oral, gastrostomy, and duodenostomy groups when no interaction is present (*p* < 0.05). Symbols of *p-value of route main effect and p*ectin main effects (LMP, HMP, and PF) are illustrated at the bottom of each panel.

Cecal microbiota composition was profiled by 16S rRNA sequencing. For alpha diversity, the Chao1 index, an indicator of species richness, showed a significant main effect of pectin type, with significantly higher values in the LMP group than in the HMP and PF groups (Figure 4A; *p* < 0.05). The Shannon diversity index also showed significant main effects of both administration route and pectin type (Figure 4B; *p* < 0.05). Specifically, Shannon index values were significantly lower in the oral and duodenostomy groups than in the gastrostomy group (Figure 4B; main effect of administration route, *p* < 0.05), and significantly higher in the LMP group than in the HMP and PF groups (Figure 4B; main effect of pectin type, *p* < 0.05). Beta-diversity analysis based on weighted UniFrac distances revealed distinct clustering according to administration route and pectin type in PCoA (Figure 4C).

Relative abundance was examined at the phylum (Figures 4D–G) and family (Figures 4H–L) levels. At the phylum level, Firmicutes accounted for more than 50% of the mean relative abundance, followed by Bacteroidetes and Proteobacteria (Figure 4D). The relative abundance of Firmicutes was significantly lower in the duodenostomy group than in the oral and gastrostomy groups (Figure 4E; main effect of administration route, *p* < 0.05) and was significantly higher in the LMP group than in the HMP group (Figure 4E; main effect of pectin type, *p* < 0.05). The relative abundance of Proteobacteria was significantly higher in the duodenostomy group than in the oral and gastrostomy groups, irrespective of pectin type (Figure 4G; interaction effect, *p* < 0.05). At the family level, Lachnospiraceae, Enterobacteriaceae, Bacteroidaceae, and Enterococcaceae showed the highest average relative abundances (Figure 4H). Lachnospiraceae abundance was lowest in the duodenostomy group, followed by the gastrostomy group, and highest in the oral group (Figure 4I; main effect of administration route, *p* < 0.05). Enterobacteriaceae abundance was highest in the duodenostomy group, followed by the gastrostomy and oral groups (Figure 4J; main effect of administration route, *p* < 0.05), and was significantly lower in the LMP group than in the HMP group (Figure 4J; main effect of pectin type, *p* < 0.05). Enterococcaceae abundance showed a significant interaction between administration route and pectin type. Across pectin types, abundance was higher in the duodenostomy group than in the oral group, and within the LMP and PF groups it was also higher than in the gastrostomy group (Figure 4L; *p* < 0.05).

### 3.4 Plasma endotoxin and indole concentrations

**Figure 5:**
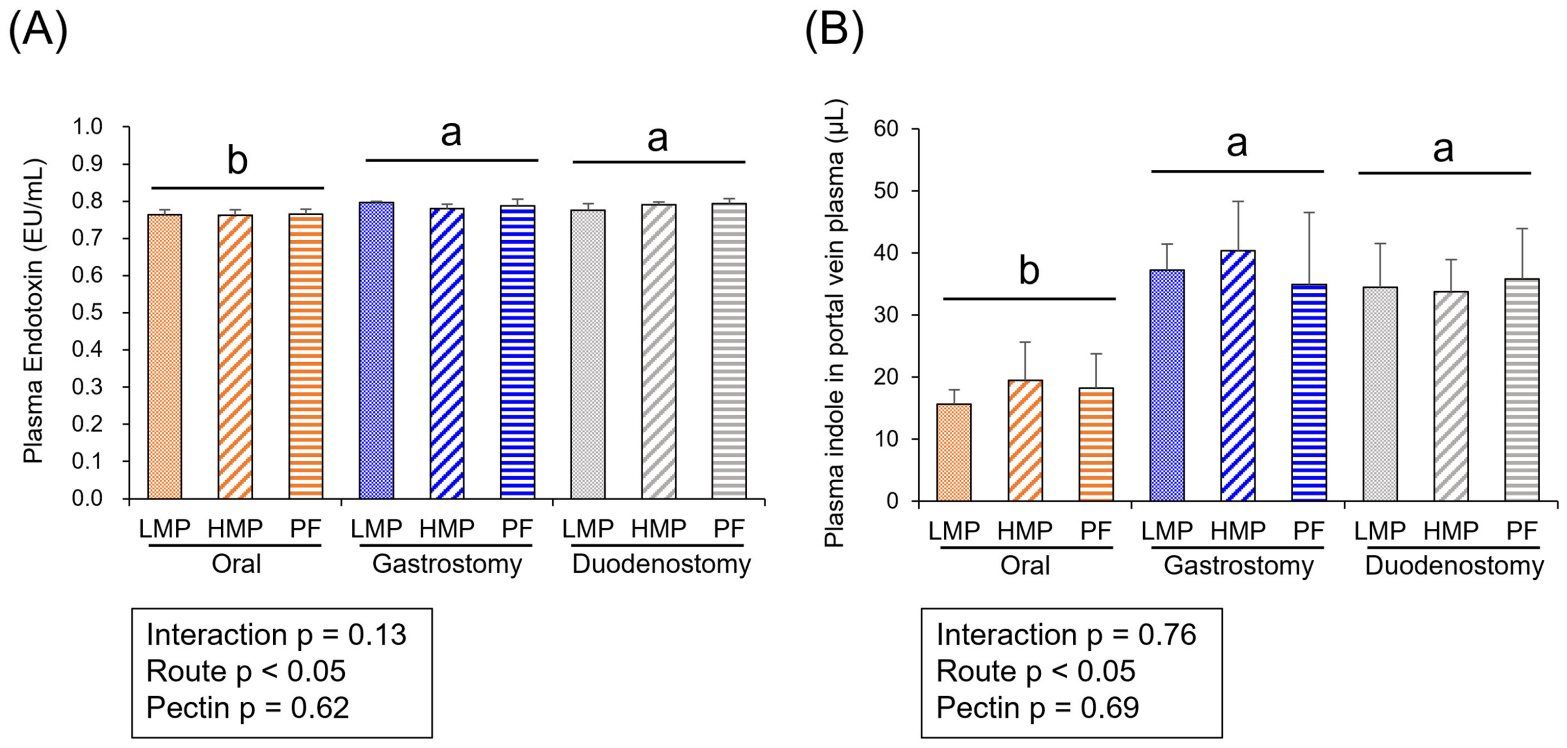
Endotoxin and indole concentrations in portal vein plasma. **(A)** Endotoxin concentration, **(B)** Indole concentration. Compared to the oral group, both indicators increased significantly in the gastrostomy and duodenostomy groups. Data are presented as mean ± standard deviation (SD) (*n* = 6 per dietary condition, *n* = 18 per administration route group or pectin group). When significant main effects or interaction effects were observed, the means were compared using Tukey’s HSD multiple comparison test (*p* < 0.05). Different letters (a, b, and c) indicate significant differences among the oral, gastrostomy, and duodenostomy groups (*p* < 0.05). Symbols of *p*-val*ue of route main effect and p*ectin main effects (LMP, HMP, and PF) are illustrated at the bottom of each panel.

Portal vein plasma endotoxin and indole concentrations were significantly higher in the gastrostomy and duodenostomy groups than in the oral group (Figure 5A,B; main effect of administration route, *p* < 0.05).

## 4 Discussion

In this study, we evaluated the effects of administration route (oral, gastrostomy, and duodenostomy) and pectin type (LMP, HMP, and PF) on physiological parameters and the gut microbiota using gastrostomy and duodenostomy rat models.

For body weight, significant main effects were observed for both administration route and pectin type (Figure 2A). Compared with the HMP and PF groups, fecal consistency was improved in the LMP group, indicating that inclusion of LMP in the liquid diet ameliorated fecal characteristics (Figure 3A). This effect may be attributed to the prevention of fecal consistency deterioration through fecal solidification associated with the water retention properties of LMP (17). This is consistent with previous reports showing that normalization of fecal consistency contributes to weight maintenance or gain (18). In contrast, liquid diets containing HMP or no pectin may have been associated with impaired nutrient absorption secondary to deterioration of fecal consistency. Although diarrhea remains a challenge in nutritional management with liquid diets (2), it has been reported that liquid diets containing LMP administered orally or via gastrostomy can normalize stool consistency (17, 18). The present study further demonstrated that LMP normalized fecal consistency even when administered via the duodenal route.

Decreased plasma albumin levels and elevated AST, ALT, and ammonia levels were observed following direct and sustained administration of liquid nutrition via the stomach or duodenum, suggesting a potential link to liver function and metabolism; however, the underlying mechanisms remain unclear. As gastric acid secretion plays a key role in iron absorption (25), the reduced plasma iron concentration observed in the duodenostomy group is likely due to impaired solubilization of dietary iron in the absence of gastric acid, leading to decreased intestinal absorption. These findings indicate that differences in administration route may influence nutrient absorption, trace element metabolism, and gastrointestinal function.

Cecal content weight is regarded as a potential indicator of intestinal transit and retention time (26–28). In this study, cecal content weight was significantly higher with duodenostomy than with gastrostomy feeding, suggesting a possible delay in intestinal transit time. In patients undergoing laparoscopic sleeve gastrectomy, administration of semi-solid labeled food has been reported to delay inflow and transit to the ileocecal region (29). Because duodenostomy bypasses gastric emptying, liquid diets are delivered directly to the intestine, which may alter intestinal motility compared with gastrostomy of oral feeding. Furthermore, cecal content weight was significantly lower in rats receiving LMP-containing liquid diets than in those receiving HMP-containing or pectin-free diets. Kagawa et al. (30) reported that, in rats, LMP-containing liquid diets shorten gastrointestinal transit time via two mechanisms: enhanced intestinal motility resulting from gelation under acidic gastric conditions and promotion of defecation associated with increased SCFA production. These results suggest that administration of an LMP-containing liquid diet reduces cecal contents, thereby shortening gastrointestinal transit time.

With respect to the gut microbiota, the Shannon diversity index was significantly lower in the duodenostomy group than in the gastrostomy group. These findings suggest that, even when the same LMP-containing liquid diet that maintained favorable fecal characteristics across routes was used, administration via duodenostomy may be associated with reduced microbial diversity. Previous studies have reported that, compared with healthy children, those with severe conditions requiring gastrostomy tubes exhibit reduced *α* diversity in their gut microbiota (16). Tottey et al. (31) further reported that prolonged intestinal transit time is associated with reduced gut microbial diversity, and Johnson-Martínez et al. (32) demonstrated that delayed intestinal transit alters the gut environment through protein fermentation and toxin production, thereby decreasing microbial diversity. Together with the increased cecal content weight observed in the duodenostomy group, these findings suggest that duodenostomy may be associated with delayed intestinal transit, leading to reduced gut microbiota diversity. In addition, administration of LMP-containing liquid diets significantly increased both the Chao1 and Shannon indices, supporting the possibility that, among dietary fibers, LMP helps maintain and promote gut microbiota diversity in rats. Beta-diversity analysis based on weighted UniFrac distances further suggests that the gut environment undergoes dynamic changes depending on administration route and pectin type.

Regarding the relative abundance of gut microbiota, the duodenostomy group showed a significant decrease in the phylum Firmicutes and a significant increase in the phylum Proteobacteria compared with the oral and gastrostomy groups. These changes highlight the influence of the nutritional administration route on the intestinal environment and suggest that direct delivery of nutrients to the intestine via duodenostomy, bypassing the stomach, may substantially alter gut microbiota composition at the phylum level. For instance, the family Lachnospiraceae, belonging to the phylum Firmicutes, contains many butyrate-producing bacteria (33). Butyrate is known to maintain intestinal barrier function (34) and exert anti-inflammatory effects (35). The relative abundance of Lachnospiraceae was markedly reduced in the duodenostomy group compared with the oral and gastrostomy groups, and was also reduced in the gastrostomy group compared with the oral group (Figure 4I), suggesting potential implications for intestinal immune regulation and inflammatory control. Nakai et al. (16) similarly reported a reduced proportion of butyrate-producing bacteria in the gut microbiota of critically ill children receiving tube feeding compared to healthy children. Taken together, these findings suggest that enteral nutrition delivered via gastrostomy or duodenostomy, in contrast to oral intake, may exert broad effects on physiological functions, including intestinal immune regulation, metabolism, and inflammatory control. An increase in the phylum Proteobacteria, including members of the family Enterobacteriaceae (e.g., Escherichia and Salmonella), is considered a marker of intestinal dysbiosis (36). Expansion of the family Enterobacteriaceae within Proteobacteria has been associated with increased inflammation and a higher risk of infection (37). In the present study, the relative abundance of Enterobacteriaceae and Enterococcaceae was significantly higher in the duodenostomy group and was also increased in the gastrostomy group compared with the oral group (Figures 4J,L). These findings suggest that bypassing the oral route for nutrient delivery may disrupt gut microbiota homeostasis, potentially leading to impaired intestinal barrier function and enhanced inflammatory responses. Conversely, administration of the LMP formula significantly increased the phylum Firmicutes and decreased Enterobacteriaceae compared with HMP. These findings indicate that differences in pectin structure and fermentability influence gut microbiota composition, and further suggest that LMP may help ameliorate intestinal environmental alterations induced by enteral nutrition via gastrostomy or duodenostomy. The changes in the gut microbiota observed in this study may reflect the effects of continuous enteral feeding on the digestion and absorption of dietary protein (31) and fiber, as well as subsequent bacterial fermentation and metabolism.

Compared with the oral group, the duodenostomy group showed increased dominance of the phylum Proteobacteria and the family Enterobacteriaceae (38), which are associated with pathogenicity and endotoxin production (Figures 4G,J). In addition, elevated portal vein plasma endotoxin and indole levels were observed in the duodenostomy group (Figure 5). Together, these findings are consistent with the possibility that enteral feeding via duodenostomy is associated with intestinal barrier dysfunction and dysbiosis. In addition, decreased plasma albumin concentrations and increased plasma AST, ALT, and ammonia concentrations were observed in the duodenostomy group (Figures 2B,D–F). These alterations may reflect changes in liver-related biochemical parameters potentially associated with conditions commonly linked to dysbiosis, including inflammatory processes (39). Previous studies have reported that inflammation-associated liver fibrosis is characterized by an increased abundance of Proteobacteria, including *Escherichia coli*, and a reduced abundance of Firmicutes (39). Notably, a similar microbial pattern was observed in the present study, which is consistent with, but does not establish, this proposed link. Additional verification using histological analyses and inflammatory markers will be required to clarify the mechanistic relationships among feeding route, gut microbiota alterations, endotoxin-related inflammation, and liver dysfunction.

This study has several limitations that should be acknowledged. First, only male rats were included; therefore, the findings are inherently restricted to a single sex. This limitation is particularly significant, as sex is a key biological variable influencing gastrointestinal physiology and host–microbiota interactions. Previous studies have demonstrated that sex differences exist in colonic transit and gut microbiota composition, mediated by factors such as sex hormones, immune function, and bile acid metabolism (40). In addition, phytoestrogens derived from soy protein, the protein source used in the liquid diet in this study, may exert endocrine and metabolic effects in both sexes via estrogen receptor signaling (41). However, because only males were examined, the present study design does not permit the evaluation of sex-dependent responses or interactions between dietary components and sex-specific pathways. Furthermore, the exclusion of females precludes the assessment of sex-specific effects, which may limit the generalizability of the findings and their relevance for clinical application (42). Therefore, the extent to which these results can be extrapolated to females or broader clinical populations remains restricted, and future studies incorporating both sexes are warranted. Second, in the oral group, animals freely consumed liquid diets, resulting in differences in feeding rate, timing, and fasting duration compared with the other administration routes. As the feeding regimens between groups varied depending on the route of administration, we cannot rule out the possibility that feeding patterns (intermittent versus continuous feeding) independently affected gastrointestinal motility, metabolic responses, and gut microbiota composition. Further experiments comparing feeding patterns (continuous administration vs. intermittent administration) are necessary to confirm whether the findings were attributable to the administration itself or the feeding regimen. Additionally, in the gastrostomy and duodenostomy groups, the nature of enteral feeding made it impossible to eliminate the chronic stress associated with the feeding method. Third, the proposed mechanistic pathway linking feeding route, gut microbiota alterations, endotoxin elevation, and liver dysfunction remains speculative. Although associations among these variables were observed, causal relationships were not directly tested, as no interventions targeting microbiota modulation or endotoxin signaling were performed. Fourth, the gut microbiota data were obtained from a rat model; therefore, it remains unclear whether similar findings would be observed in humans, whose gut microbiota composition differs substantially. A comprehensive study is needed to clarify how differences in clinical administration routes affect the human gut microbiota and physiological responses (43).

## 5 Conclusion

Using a rat model, this study systematically compared the effects of enteral feeding route (oral, gastrostomy, and duodenostomy) and pectin type (LMP, HMP, and PF) on fecal characteristics and gut microbiota composition. The results show that, compared to gastrostomy feeding, duodenal tube feeding increased cecal content weight and decreased gut microbiota diversity. It also induced compositional changes in the gut microbiota, including a reduction in the phylum Firmicutes and an increase in the phylum Proteobacteria, compared with oral or gastrostomy feeding. In contrast, LMP-containing liquid diets were associated with favorable fecal characteristics across administration routes and showed significantly higher alpha-diversity indices (Chao1 and Shannon) than HMP-containing and PF diets. Furthermore, LMP increased the abundance of the phylum Firmicutes and decreased the relative abundance of inflammation-associated bacteria (e.g., the family Enterobacteriaceae) compared with HMP, suggesting the potential of LMP to correct dysbiosis caused by enteral nutrition.

These findings suggest that the route of liquid diet administration is associated with the intestinal environment and that these effects vary depending on the type of dietary fiber. The present results provide experimental evidence of route and fiber-dependent associations and may help generate hypotheses for future translational and clinical studies, rather than supporting direct clinical recommendations.

## Data Availability

The data presented in the study are deposited in the Mendeley Data repository, DOI: 10.17632/x3nk6m4tb5.1.
